# Epidemiological Significance of SARS-CoV-2 RNA Dynamic in Naso-Pharyngeal Swabs

**DOI:** 10.3390/microorganisms9061264

**Published:** 2021-06-10

**Authors:** Paolo Calistri, Maria Luisa Danzetta, Laura Amato, Francesca Cito, Alessandra Di Giuseppe, Valentina Zenobio, Daniela Morelli, Ilaria Puglia, Marialuigia Caporale, Silvia Scialabba, Giovanni Savini, Giacomo Migliorati, Nicola D’Alterio, Alessio Lorusso

**Affiliations:** Istituto Zooprofilattico Sperimentale dell’Abruzzo e del Molise G. Caporale, 64100 Teramo, Italy; p.calistri@izs.it (P.C.); m.danzetta@izs.it (M.L.D.); l.amato@izs.it (L.A.); f.cito@izs.it (F.C.); v.zenobio@izs.it (V.Z.); d.morelli@izs.it (D.M.); i.puglia@izs.it (I.P.); mr.caporale@izs.it (M.C.); s.scialabba@izs.it (S.S.); g.savini@izs.it (G.S.); g.migliorati@izs.it (G.M.); n.dalterio@izs.it (N.D.); a.lorusso@izs.it (A.L.)

**Keywords:** SARS-CoV-2, cycle threshold, Abruzzo, COVID-19, naso-pharyngeal swab, viral circulation

## Abstract

From 16 March to 15 December 2020, 132,357 naso-pharyngeal/oropharyngeal swabs were collected in the province of Teramo, Abruzzo Region, Italy, and tested for the presence of SARS-CoV-2 genomic RNA by a commercially available molecular assay. A total of 12,880 swabs resulted positive. For 8212 positive patients (4.150 women and 4.062 men) the median age was statistically different between women (median: 49.55 ± 23.9 of SD) and men (median: 48.35 ± 23.5 of SD) while no differences were found in the comparison between the cycle threshold for the N protein-encoding gene (C*_T_* N) median values and gender. Differences were observed in the C*_T_* N gene median values of swabs collected from March to September as well as in the pairwise comparison between September and October and between November and December. The C*_T_* N gene median values observed in specific periods characterizing the SARS-CoV-2 epidemic in 2020 were also compared with the incidence of COVID-19 cases; a strong inverse correlation was highlighted (Pearson correlation coefficient = −0.978). Our findings confirm the usefulness of the C*_T_* N values as an indirect detection parameter to monitor viral loads in the population.

## 1. Introduction

Since the emergence of SARS-CoV-2, almost 137,866,311 million confirmed cases of COVID-19 cases were reported globally [1] (up to 16 April 2021). In Italy, a total of 3809,193 million cases were confirmed (up to 16 April 2021), with 115,557 reported fatalities [2]. In order to support the national healthcare system, the Ministry of Health appointed the Istituti Zooprofilattici Sperimentali, public health veterinary institutes, to test for the presence of SARS-CoV-2 RNA, naso-pharyngeal/oropharyngeal swabs (NNS) collected from suspected human cases [3,4].

Accordingly, starting from 16 March 2020, swabs collected in the province of Teramo from Abruzzo, a central region of Italy, are regularly tested for the presence of SARS-CoV-2 RNA at the Istituto Zooprofilattico Sperimentale dell’Abruzzo e del Molise (IZSAM).

In a preliminary study conducted in 2020 [4], the mean of cycle threshold for the N protein-encoding gene (C*_T_* N) values of the first swab from each positive patient tested from March to May 2020, was analyzed. In that study, we observed a trend of C*_T_* increasing from a median value of 27.9 (±6.0 C*_T_* median value of standard deviation) to 33.43 (±1.3 C*_T_* median value of standard deviation) between March and May 2020, suggesting that lockdown measures and the elimination of infection foci were able to reduce the overall viral loads [4]. In that period, a general lockdown was applied from March 9 until May 18 to the whole of Italy, allowing a significant reduction of virus transmission [5]. Furthermore, a statistically significant reduction of viral RNA loads was observed in the pairwise comparison between swabs collected in April and March 2020 [4]. In contrast, the absence of a statistically significant difference between May and April 2020 was probably due to the low number of positive samples detected in May [4]. In addition, no differences between C*_T_* N gene values were observed between males and females [4].

**Figure 1:**
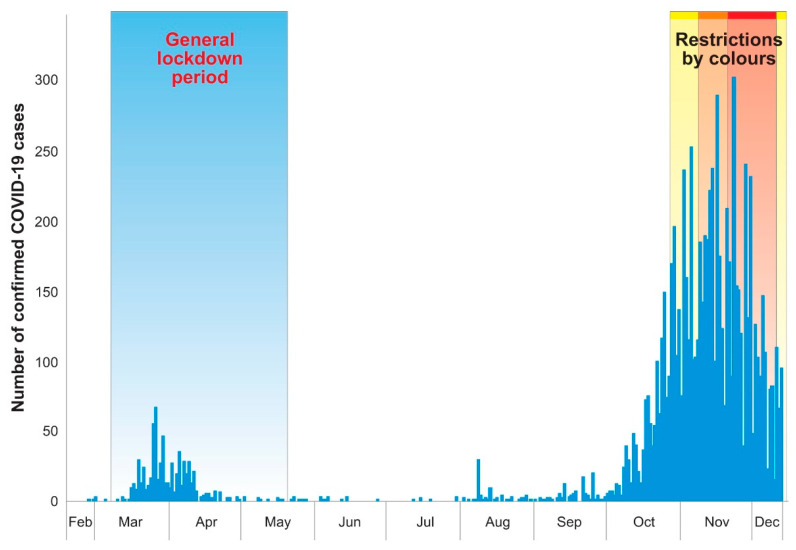
Number of COVID-19 cases in Teramo province per day throughout 2020. The first period of national lockdown from 9 March 2020to 18 May 2020is highlighted. From October 2020 a system modulating the restrictions on mobility based on colours (increasing the levels of restriction from yellow to red) was established. and highlighted.

In the first three months of the epidemic, from March 2020 to the end of May, a total of 660 COVID-19 cases were confirmed in the province of Teramo [2]. Likewise, other Italian provinces, following a summer characterised by limited viral circulation, in September a rapid increasing of the epidemic curve was observed in the province of Teramo, which was overwhelmed by 7814 confirmed cases from October to December 2020 (Figure 1) [2].

Following the results of the preliminary study [4], further analysis of the C*_T_* N gene values of positive NNS swabs in patients tested from March to 15 December 2020 was performed in the province of Teramo in order to characterize the dynamic of SARS-CoV-2 RNA loads over a longer period of time and to verify the existence of links between C*_T_* N gene values and the epidemiological situation in the area.

The analysis was limited up to 15 December 2020 in order to avoid the interference with the B.1.1.7 lineage (VOC 202012/01) of SARS-CoV-2 positive cases detected for the first time in Abruzzo in that period [6].

## 2. Materials and Methods

The workflow for SARS-CoV-2 RNA detection in NNS swabs [3] has been previously described [3,4]. Briefly, the adopted molecular test (TaqMan 2019-nCoV (qPCR) assay kit v2 manufactured by Thermo Fisher (Thermo Fisher Scientific, Waltham, MA, USA) targets three different portions of SARS-CoV-2 genome namely the replicase, S and N protein-encoding genes. For practical reasons, out of the three values produced for each sample, for the downstream analysis, only the C*_T_* value associated with detection of the N protein-encoding gene was selected, since it is translated by the most abundant viral sub-genomic RNA [7].

Statistical analysis was performed using StatTools© (version 7.5.2 Palisade Corporation, Ithaca, NY, USA). A Mann–Whitney test was used to assess the statistical significance of differences among the C*_T_* N gene median values in NNS swabs collected from March to 15 December 2020 in the province of Teramo. Regression analysis was performed to verify the correlation between C*_T_* N gene median values and the incidence per 10,000 inhabitants of COVID-19 cases registered in three specific periods of the year: from March to April, from May to September and from October to December. These observation periods were selected because they characterize the epidemic curve observed in Italy: the first epidemic wave, the inter-epidemic period, and the second epidemic wave (Figure 1).

For each SARS-CoV-2 positive patient, the C*_T_* N gene value of the first positive swab was considered. The level of significance was set at a p-value of 0.05. In order to check the performance of the swabbing procedure, a real-time RT-PCR detecting the human ribonuclease P RNA component H1 (H1RNA) gene (RPPH1) on chromosome 14 was employed. This molecular test, included in the TaqMan 2019 nCoV Assay kit v1 by Thermo Fisher, was run with a total of 68 randomly selected SARS-CoV-2 RNA positive samples, within those included in this study. The swabs were selected, in equal number, based on the C*_T_* N value, including: C*_T_* N between 15 and 20 (C*_T_* N 15-20), C*_T_* N 20-25, C*_T_* N 25-30, and C*_T_* N 30-35. Furthermore, in order to assess the presence of a correlation between C*_T_* values and viral loads, log_10_ dilutions (seven replicates per dilution) of a SARS-CoV-2 isolate on cell cultures were processed by qPCR.

## 3. Results

From 16 March to 15 December 2020, IZSAM analyzed a total of 132,357 NNS collected in the province of Teramo and 12.880 NNS tested positive for SARS-CoV-2 RNA by qPCR. A total of 8212 positive patients was selected for further analysis, of which 4150 women and 4062 men (Table 1). The median age (years) was found statistically different (two-tailed Mann–Whitney test, *p*-value < 0.01) between women (median: 49.55 ± 23.9 of standard deviation) and men (median: 48.35 ± 23.5 of standard deviation). The C*_T_* N gene median values of the first positive swab between women and men were calculated and no statistically significant differences (two-tails Mann–Whitney test, *p*-value > 0.05) were found between women (median C*_T_* N: 29) and men (median C*_T_* N: 28).

**Figure 2:**
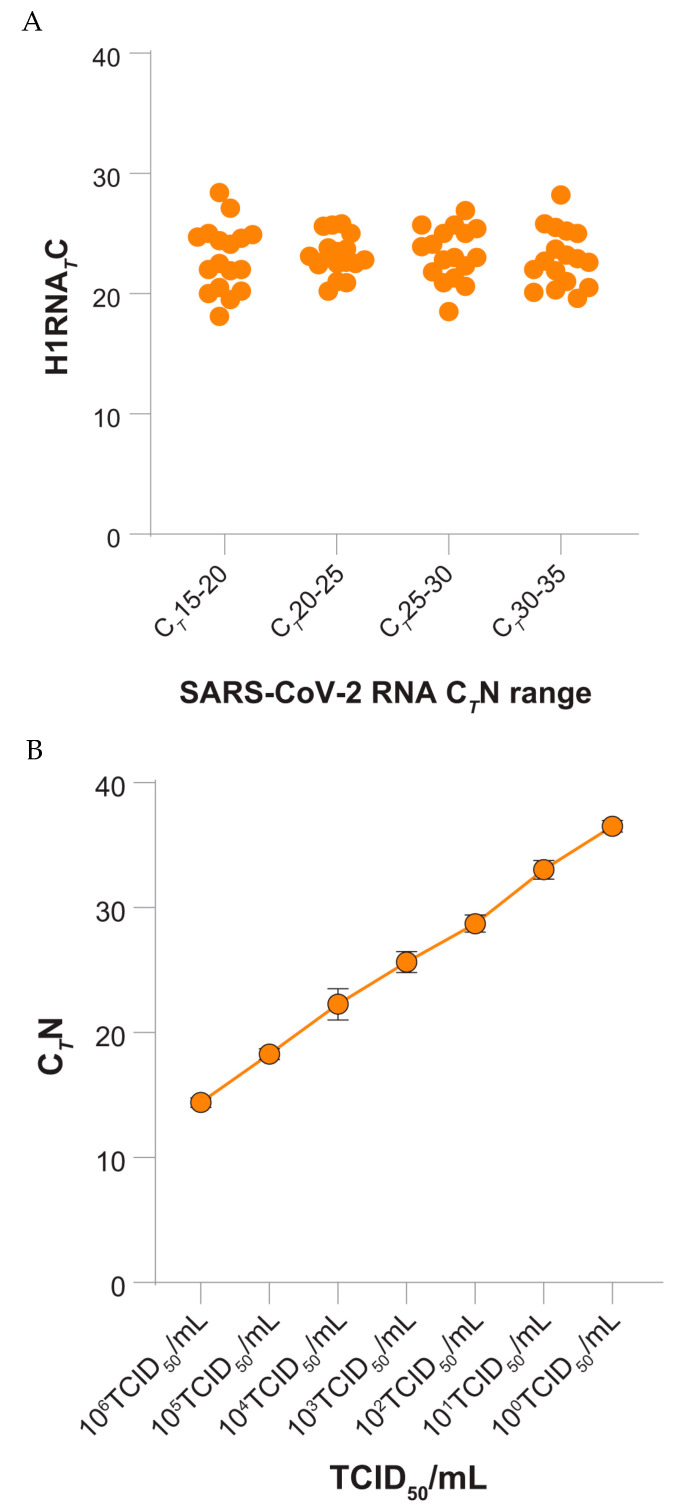
(**A**) Individuals C*_T_* N values of H1RNA of SARS-CoV-2 RNA positive samples (*y* axis). A number of 68 SARS-CoV-2 RNA positive samples were selected according to the C*_T_* N value and divided in four groups (*x* axis), (**B**) Mean with standard deviation of C*_T_* N values (*y* axis) of seven dilution of a SARS-CoV-2 isolate (*x* axis). Each dilution was tested in seven replicates. TCID, tissue culture infectious dose.

C*_T_* values of the H1RNA gene, although reasonably analysed on a selected set of samples, clearly showed that the observed trends of SARS-CoV-2 C*_T_* N values are not related to the swabbing procedure as C*_T_* values of the H1RNA are constant (mean range 22.94 ± 2.794 SD to 23.29 ± 2.28 SD) in samples showing different loads of SARS-CoV-2 RNA (Figure 2A). 

In order to assess the presence of a correlation between C*_T_* values and viral loads, log_10_ dilutions (seven replicates per dilution) of a SARS-CoV-2 isolate on cell cultures were processed. The results showed a linear correlation over seven order of magnitude (from 10^6^ TCID_50_/mLto 10^0^ TCID_50_/mL), with good intra-assay reproducibility at high, intermediate and low virus concentrations (Figure 2B).

**Figure 3:**
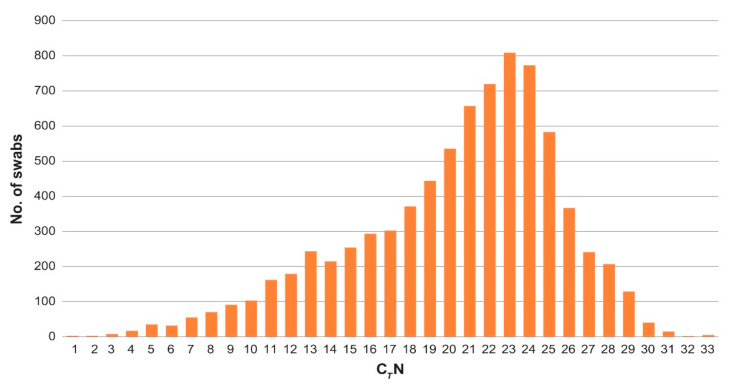
Distribution of the C*_T_* N values in the 8212 positive NNS considered in the study.

Concerning the C*_T_* N values observed in the 8212 positive NNS, the mean and median values were 27.59 (±0.0983 CI 90%) and 29.00, respectively (Figure 3).

Furthermore, the C*_T_* N gene median values of the first positive swab for each individual were compared over time. In Table 2 the C*_T_* N gene values obtained during the months of observations and the number of swabs considered for each month are reported. 

**Figure 4:**
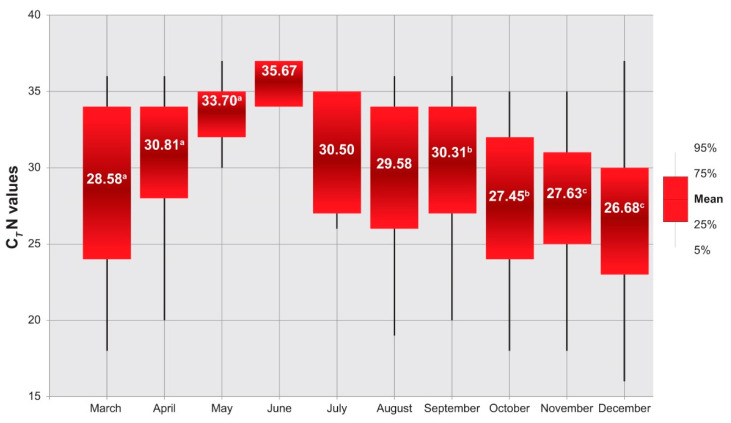
Mean values of C*_T_* N gene of the first positive swab per patient by month. Letters are denoting a statistically significant difference between values of C*_T_* N gene (*p*-value < 0.05).

A statistically significant difference (two-tailed Mann-Whitney test) was observed among the C*_T_* N gene median values of swabs collected from March to September (Figure 4). In particular, the C*_T_* N gene median values observed in NNS collected in March 2020 were significantly lower than those collected in April (*p*-value < 0.0001), and the latter lower than those collected in May 2020 (*p*-value = 0.0033), thus suggesting progressively lower viral RNA loads from March to May 2020. A significant difference was also observed in the pairwise comparisons between September and October 2020 (*p*-value < 0.0001) and between November and December (*p*-value < 0.0001).

When the median values of C*_T_* N observed in the three main periods (March-April, May-September and October-December) characterising the SARS-CoV-2 epidemic in 2020 were compared with the incidence of COVID-19 cases (Table 3), a strong inverse correlation (Pearson correlation coefficient = −0.978) was observed.

In turn, no correlation between the C*_T_* N values and the age of the infected individuals was observed (Pearson correlation coefficient = −0.094).

## 4. Discussion

Although RNA loads in swabs can be influenced by several factors including the clinical status of the patients, timing and swabbing procedure, we analyzed the trend of the C*_T_* N values in NNS collected from March to mid-December 2020. 

In this study, we assumed that albeit not a good predictor of viral load when looking at an individual patient’s test results [8], C*_T_* values may give an indirect indication of the general viral load in the exposure environment when the population level is considered. This kind of approach has been already and successfully applied in other studies [9,10], and C*_T_* values have been used not only as proxies for the amount of virus circulating in the population under study, but also to predict the trajectory of the epidemic [10]. Although measured by diagnostic molecular method detecting genomic RNA rather than only subgenomic RNAs or virus isolation, analysis of viral load may indeed provide critical data to implement effective control measures and disease modeling [11]. 

A pattern with progressive increasing of C*_T_* N gene values was observed from March to May, testifying to an overall reduction of viral RNA load in swabs. This pattern was inverted from September to October and then from November to December, with an increase of viral RNA load in swabs. It is also important to point out that this analysis was limited up to 15 December 2020. This was done to avoid the effect of B.1.1.7 lineage (VOC 202012/01) of SARS-CoV-2 firstly identified in mid-December 2020 in the Abruzzo region. Before that period, indeed, circulation of SARS-CoV-2 lineages belonging to variants of concern (e.g., B.1.1.7, P.1, and B.1.351) was not evidenced. Specifically, lineage B.1.177 (also known as Spanish variant) emerged in summer 2020 and quickly became the dominant lineage in this area during fall as a result of opening borders in summer 2020. 

The first reduction of viral RNA load was observed from March to May, and it was concurrent with the effect of the general lockdown established all over Italy, with analogous repercussions also in the Teramo province (Figure 3). 

Accordingly, the observed increase of RNA loads in NNS tested from September onward could be linked to the general relaxation of preventive measures during the summer as a result of the cessation of the general lockdown in Italy. This link is confirmed by the strong inverse correlation between + median values and COVID-19 incidence per 100,000 inhabitants, when the three main epidemiological periods, characterized by different rates of COVID-19 incidence, are considered. As highlighted in our previous work [4], one of the pitfalls of our findings is the lack of anamnestic data for each positive individual. Detailed information for each patient on the clinical status prior and after SARS-CoV-2 infection, disease severity, the date of symptom’s onset would be beneficial to better interpret the dynamic of RNA loads in NNS.

The data originated in this analysis are supported by the evidence that SARS-CoV-2 C*_T_* N value trends are not affected by the swabbing procedure as the human H1RNA gene, used as control, was constant across a selected set of SARS-CoV-2 positive samples showing a wide range of viral loads. Moreover, a correlation was evidenced between C*_T_* values and infectious virus in vitro, therefore high C*_T_* values (>30) are likely predictive of very limited amounts of infectious virus (≤10^1^ TCID_50_/mL). 

In conclusion, our findings strongly confirm the usefulness of using qPCR and related C*_T_* values as an indirect mean for monitoring the levels of viral load circulation in the population, although the emergence of new variants of the virus at the end of 2020, apparently more transmissible than the others, is posing new questions that should be addressed in the early future.

## Figures and Tables

**Table 1:** Number of individuals with a positive NNS in the province of Teramo included in the study (16 March–15 December 2020).

Age (Years)	Women	Men	Total
0–10	209	259	468
11–20	427	463	890
21–30	397	372	769
31–40	470	489	959
41–50	614	541	1155
51-60	756	681	1437
61–70	475	551	1026
71–80	324	363	687
81–90	278	250	528
91–100	188	85	273
>100	12	8	20
Total	**4150**	**4062**	**8212**

**Table 2:** Values of C*_T_* N gene of the first positive swab observed in each month.

C*_T_* N values								
Month	Mean	Median	Minimum	Maximum	Standard Deviation	5th percentile	95th Percentile	Number of Swabs
March	28.6	30.0	12.0	40.0	6.0	18.0	36.0	316
April	30.8	32.0	14.0	40.0	4.9	20.0	36.0	202
May	33.7	34.0	27.0	40.0	2.5	30.0	37.0	27
June	35.7	36.0	34.0	37.0	1.5	34.0	37.0	3
July	30.5	30.0	26.0	35.0	4.0	26.0	35.0	6
August	29.6	31.0	18.0	36.0	5.1	19.0	36.0	59
September	30.3	32.0	18.0	38.0	4.9	20.0	36.0	96
October	27.4	28.0	11.0	39.0	5.5	18.0	35.0	1501
November	27.6	29.0	8.0	38.0	5.1	18.0	35.0	4339
December	26.7	28.0	8.0	38.0	5.7	16.0	37.0	1663

**Table 3:** Median values of C*_T_* N gene of the first positive swab, number of COVID-19 cases and incidence per 100,000 inhabitants observed in three periods of 2020 (March-April, May-September and October-December) in the province of Teramo.

	Median C*_T_* N Value	COVID-19 Cases	Incidence (×100,000)
March–April	31	641	208.12
May–September	32	373	121.10
October–December	28	7814	2537.01

## Data Availability

Data sharing not applicable.
